# Quantitative Morphological Profiling and Isolate-Specific Insensitivity of Cacao Pathogens to Novel Bio-Based Phenolic Amides

**DOI:** 10.3390/jof12010033

**Published:** 2026-01-01

**Authors:** Ezekiel Ahn, Masoud Kazem-Rostami, Sunchung Park, Richard D. Ashby, Helen Ngo, Lyndel W. Meinhardt

**Affiliations:** 1 Sustainable Perennial Crops Laboratory, Agricultural Research Service, United States Department of Agriculture, Beltsville, MD 20705, USA; 2 Sustainable Biofuels and Co-Products Research Unit, Agricultural Research Service, United States Department of Agriculture, Eastern Regional Research Center, Wyndmoor, PA 19038, USA

**Keywords:** *Colletotrichum gloeosporioides*, *Pestalotiopsis*, quantitative morphological profiling, bio-based compounds, machine learning, isolate specificity

## Abstract

Fungal pathogens, including *Colletotrichum gloeosporioides* and *Pestalotiopsis* spp., are significant threats to global cacao production. Understanding their varying responses to novel antifungal agents is crucial for developing sustainable plant protection strategies. This study investigated the quantitative morphological responses and isolate-specific sensitivity of three cacao pathogen isolates (one *Pestalotiopsis* sp. and two *C. gloeosporioides*) to four novel bio-based phenolic-branched fatty acids and their corresponding amides derived from renewable feedstocks. We observed a high degree of isolate-specific susceptibility. A phenol-branched soy oil-derived fatty amide (PhSOAM) proved most potent, significantly inhibiting the growth of *Pestalotiopsis* sp. and one *C. gloeosporioides* isolate. In contrast, the second *C. gloeosporioides* isolate displayed complete insensitivity to all tested compounds, highlighting significant intraspecific variation. Notably, quantitative image analysis revealed that PhSOAM uniquely altered fungal colony morphology by significantly increasing the length-to-width ratio, suggesting a mechanism of action involving the disruption of polarized growth. Multivariate analyses and machine learning models (R^2^ up to 0.74) effectively classified these responses, identifying the specific pathogen-compound pairing as the most critical determinant of the interaction outcome. This work not only highlights the potential of bio-based amides but also establishes a powerful analytical framework, combining morphological profiling with predictive modeling, to gain deeper insights into the complex, isolate-specific nature of fungal–antifungal interactions.

## 1. Introduction

Fungal pathogens represent a persistent threat to global food security and ecosystem stability, with pathogens of cacao (*Theobroma cacao*) being particularly devastating [1]. As the source of chocolate, cacao is a cornerstone of many developing economies, supporting the livelihoods of millions of smallholder farmers [2,3]. However, cacao production is severely constrained by a range of fungal diseases, including anthracnose, caused by *Colletotrichum* species, and leaf blight, caused by *Pestalotiopsis* species, which can have significant local impacts on crop yield and quality worldwide [4,5,6,7]. While synthetic fungicides have been a primary control strategy, their widespread use has led to the emergence of resistant fungal strains, creating an urgent need for novel, sustainable antifungal strategies with durable efficacy against developing resistance in key pathogens [8,9,10].

Plants naturally defend against fungal attacks by synthesizing a diverse array of phenolic compounds through the phenylpropanoid pathway [11,12,13]. These molecules, along with fatty acid amides from various natural sources, represent a promising source of leads for new fungicides [14,15,16,17]. However, the practical application of natural products can be limited by factors such as volatility or suboptimal stability. To overcome these limitations, synthetic derivatization has been employed to enhance the physicochemical properties and bioactivity of natural scaffolds [18,19,20,21]. Following this bio-based approach, we recently developed methods for branching natural phenolic compounds onto non-volatile fatty acid chains derived from renewable feedstocks, such as vegetable oils and waste greases, creating new families of phenolic-branched fatty acids and amides with proven antimicrobial efficacy [22,23,24]. The introduction of amide groups, in particular, was hypothesized to alter interactions with microbial cellular targets (e.g., disrupting membrane organization or lipid rafts), a concept supported by research into fatty acid amides and fungal sphingolipids as antifungal targets [14,20,25,26]. While this study utilized technical-grade mixtures (C16/C18-rich), preventing a strict chain-length comparison, the robust activity of soybean-derived compounds (predominantly C18) suggests that the long alkyl chains provide the necessary lipophilicity to facilitate membrane insertion, acting as an essential hydrophobic anchor for the active phenolic moiety.

Despite the potential of such derivatives, a significant challenge in antifungal discovery is understanding a compound’s mechanism of action (MOA) and spectrum of activity relative to fungal diversity [15]. Fungi exhibit remarkable phenotypic plasticity, enabling them to adapt to various niches and stresses, which can result in significant variation in drug sensitivity even among isolates of the same species [27,28]. Furthermore, the regulatory networks that control fungal development and morphogenesis are often intertwined with those that govern stress responses and drug tolerance [29]. Therefore, moving beyond simple growth inhibition assays to gain detailed insights into how specific chemical structures affect fungal morphology and isolate-specific susceptibility is critical for identifying promising leads for future agrochemical development.

**Figure 1:**
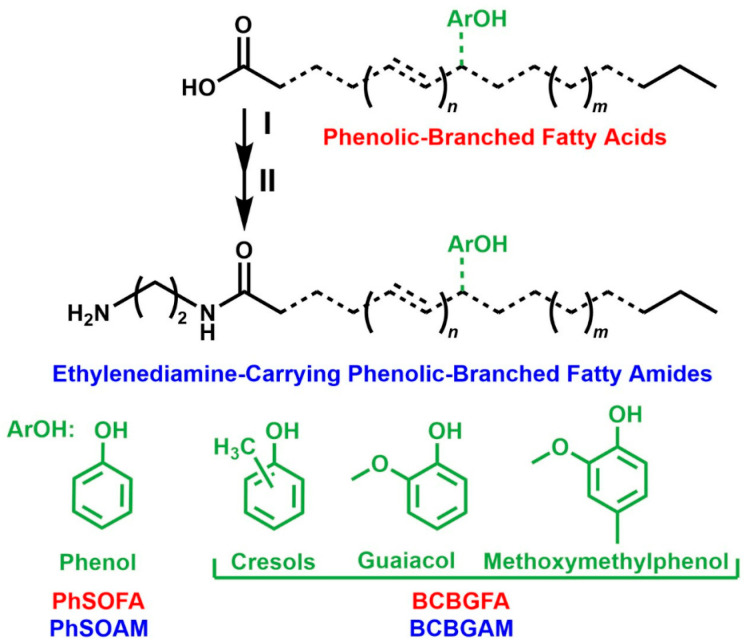
Chemical structures of the phenolic-branched fatty acids and amides studied. Chemical structures of soybean oil (SO) and brown grease (BG) fatty acids (FAs) and ethylenediamine-carrying fatty amides (AMs), whose level of unsaturation, carbon chain length, and possible isomeric variations are displayed by *n* (=0 or 1), *m* (=3 or 5), and dashed bonds, respectively. (I) Sulfuric acid catalyzed esterification in methanol at 70 °C/2 h. (II) Amidation in ethylenediamine at 160 °C/3 h. Phenolic branches (ArOH), made of phenol (Ph) and beechwood creosote (BC).

To this end, this study investigates the quantitative morphological responses and isolate-specific sensitivity of three cacao-derived fungal isolates (*Pestalotiopsis* sp. CGH5, and *C. gloeosporioides* CGH17 and CGH49) to a panel of four novel phenolic-branched fatty acids and their corresponding ethylenediamine-carrying fatty amides (Figure 1). Unlike traditional screenings, we combined in vitro morphological profiling with a predictive machine learning model to identify the key drivers of fungal susceptibility, aiming to elucidate the complex, isolate-specific interactions between these novel bio-based compounds and key cacao pathogens.

## 2. Materials and Methods

### 2.1. Biobased Compounds

The four biobased compounds tested were synthesized using renewable, technical-grade feedstocks according to previously established protocols [22,23]. In brief, the synthesis involved a two-stage process designed to valorize waste streams. First, fatty acids derived from renewable feedstocks (soybean oil for PhSOFA/PhSOAM; brown grease for BCBGFA/BCBGAM) were branched with phenolic compounds (phenol for PhSOFA/PhSOAM; beechwood creosote for BCBGFA/BCBGAM) via zeolite-catalyzed arylation to produce phenolic-branched fatty acids (PhSOFA and BCBGFA). Second, to explore the structure–activity relationship (SAR) and the functional role of the amide moiety, these fatty acids were converted to their corresponding ethylenediamine-carrying fatty amides (PhSOAM and BCBGAM) via amidation. This derivatization was strategically designed to reduce the volatility of the parent phenolic compounds and to introduce amine and amide functionalities, which are often associated with enhanced interaction with fungal cell membranes. For this study, each of the four final compounds (200 mg) was dissolved in pure ethanol to create stock solutions of 10 mg/mL prior to application.

### 2.2. Fungal Isolates and Culture Conditions

Three fungal isolates, previously obtained from diseased cacao plants in Ghana [30,31], were used in this study. The isolates were: CGH5 (*Pestalotiopsis* sp.), a causal agent of leaf blight; and CGH17 and CGH49 (*Colletotrichum gloeosporioides*), causal agents of anthracnose. All fungal isolates were maintained on Potato Dextrose Agar (PDA) medium. For experimental assays, plates were incubated in the dark at 24 °C to promote fungal growth for 7 days.

### 2.3. Antifungal Activity Assay

The antifungal effects of the four compounds were evaluated using a modified agar plate surface-coating assay. This method was chosen to simulate surface contact exposure. Each compound (10 mg/mL stock) was spread evenly across the surface of solidified PDA plates (standard 90 mm Petri dishes containing 20 mL of medium) using a sterile spreader. An aliquot of 15 µL was applied to yield a total amount of 150 µg per plate. Solvent control plates received 15 µL of pure ethanol (the same volume of the carrier solvent used for the treatment groups). A 5 mm mycelial plug from the growing edge of a 7-day-old culture of each fungal isolate was placed at the center of the treated plates. Cultures were incubated at 24 °C in the dark for 96 h. This incubation period was specifically selected to assess colony morphology during the unrestricted logarithmic growth phase, ensuring that colonies did not physically contact the plate edge, which would otherwise artificially alter shape metrics (e.g., circularity and LWR) essential for the quantitative analysis. Each isolate-treatment combination was performed with at least three biological replicates.

### 2.4. Image Acquisition and Quantitative Morphological Profiling

Following 96 h of incubation, high-resolution digital images of the fungal colonies were acquired using a Nikon Z8 camera fitted with a Nikkor Z 20 mm f/1.8 S lens (Nikon, Tokyo, Japan), mounted on a Kaiser R2N CP stand (Kaiser Fototechnik, North White Plains, NY, USA). Images were captured under consistent LED lighting conditions to ensure reproducibility. Colony morphology was quantified using SmartGrain software (version 1.1) [32]. Although primarily designed for seed phenotyping, we adapted this software for fungal analysis due to its precision in capturing contour irregularities. The outline of each colony was manually traced to strictly define the region of interest. SmartGrain then automatically calculated the following morphological traits: colony area (mm^2^), perimeter (mm), length (mm), width (mm), length-to-width ratio (LWR), circularity (a dimensionless value ranging from 0 to 1, where 1 represents a perfect circle), and the distance between the intersection of the length/width axes (IS) and the colony’s center of gravity (CG) (IS & CG, in mm), which serves as a metric for colony asymmetry.

### 2.5. Statistical Analysis and Machine Learning

All statistical analyses were performed using JMP Pro 17 (SAS Institute Inc., Cary, NC, USA) [33]. To assess differences in morphological traits between treatment groups (control, PhSOFA, PhSOAM, BCBGFA, and BCBGAM) within each fungal isolate, pairwise *t*-tests were conducted. To visualize the global morphological landscape, Principal Component Analysis (PCA) was performed using standardized data of the measured morphological traits. Multidimensional Scaling (MDS) was conducted to visualize dissimilarities between samples based on their morphological profiles. Hierarchical cluster analysis was performed using Ward’s linkage method to group both samples and traits based on similarity metrics.

For predictive modeling, we utilized JMP Pro’s Model Screening platform to evaluate multiple machine learning algorithms and identify the most effective predictor of fungal colony area. Predictor variables included fungal isolate, treatment type, LWR, circularity, and IS & CG. Models were trained and validated using 5-fold cross-validation (k = 5) with a random seed of 1. The Neural Boosted model [NTanH(3)NBoost(20)] [34], which exhibited the highest R-squared value on the validation set, was selected for detailed feature importance analysis using the “Splits”, “Sum of squares,” and “Portion” metrics.

## 3. Results

### 3.1. Antifungal Effects on Pestalotiopsis sp. (CGH5)

**Figure 2:**
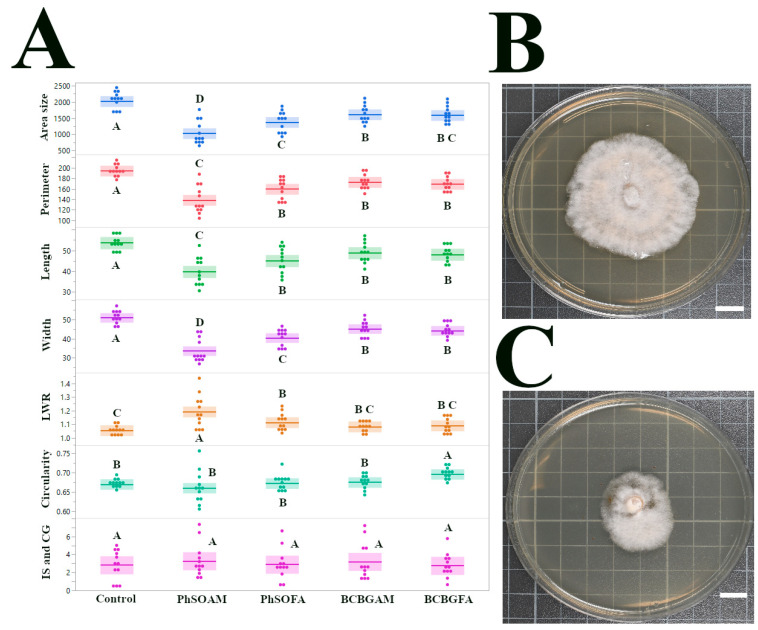
Assessment of the antifungal effects of the tested compounds on *Pestalotiopsis* sp. (CGH5): (**A**) Dot plots with solid lines indicating the mean and the interquartile range (25th to 75th percentiles), displaying the distribution of seven morphological traits: colony area size (mm^2^), perimeter (mm), length (mm), width (mm), LWR, circularity (ranging from 0 to 1, where 1 indicates a perfect circle), and IS & CG (mm). Data are based on 12 samples per treatment, with statistically significant differences (*p* < 0.05) marked by different letters above the plots, determined by pairwise *t*-tests. Non-overlapping box plots suggest potential differences, but significance is confirmed by the letters. (**B**) Representative image of a CGH5 colony after 96 h of growth on a control (ethanol-treated) PDA plate. (**C**) Representative image of a CGH5 colony after 96 h of growth on a PDA plate treated with PhSOAM, showing a notable reduction in colony size and altered morphology. White scale bars in (**B**,**C**) indicate 1 cm.

The effects of tested phenolic branched fatty acids (PhSOFA and BCBGFA) and phenolic branched fatty amides (PhSOAM and BCBGAM) on the growth of *Pestalotiopsis* sp. (isolate CGH5) are shown in Figure 2. Among the tested compounds, PhSOAM demonstrated potent growth inhibition and morphological alteration of *Pestalotiopsis* sp. (CGH5), exhibiting a striking ability to suppress fungal growth. The average colony area of the control group (CGH5 grown on untreated PDA) was 2018.53 mm^2^ (±68.46 mm^2^ SEM; *n* = 12). In a dramatic contrast, the PhSOAM treatment reduced the average colony area to a mere 1032.80 mm^2^ (±108.13 mm^2^ SEM; *n* = 12), a 48.8% reduction in fungal growth.

While the phenolic branched fatty acid PhSOFA also showed some growth-inhibitory activity, resulting in a mean colony area of 1375.81 mm^2^ (±87.02 mm^2^ SEM; *n* = 12), its effect was less pronounced than that of PhSOAM. The other two compounds, BCBGAM and BCBGFA, showed the weakest inhibition, with average colony areas of 1612.28 mm^2^ (±71.71 mm^2^ SEM; *n* = 5–10) and 1587.63 mm^2^ (±70.84 mm^2^ SEM; *n* = 12), respectively. There was no statistically significant difference in growth inhibition between BCBGAM and BCBGFA (*p* > 0.05). These observations suggest that the chemical nature of the phenolic ingredients is critical to the antifungal effect. The complex mixture of substituted phenols in beechwood creosote (e.g., guaiacol, cresols) appears to be less effective than the unsubstituted phenol ring, a finding consistent with previous antimicrobial studies [23].

Consistent with the reduction in colony area, other size-related traits (perimeter, length, and width) were also most significantly inhibited by PhSOAM (Figure 2A), demonstrating its superior efficacy in controlling the growth of the CGH5 isolate. Beyond reducing fungal growth, PhSOAM significantly altered the shape of the fungal colonies. The LWR, a measure of colony elongation, was 1.19 for the PhSOAM-treated colonies, compared to 1.05 for the control. This dramatic difference, indicating a more elongated growth pattern with PhSOAM, was highly statistically significant (*p* < 0.0001) based on a pairwise *t*-test across all replicates. The morphological shift may suggest that PhSOAM is acting through a distinct mechanism compared to the other compounds tested.

### 3.2. C. gloeosporioides Isolate CGH17

**Figure 3:**
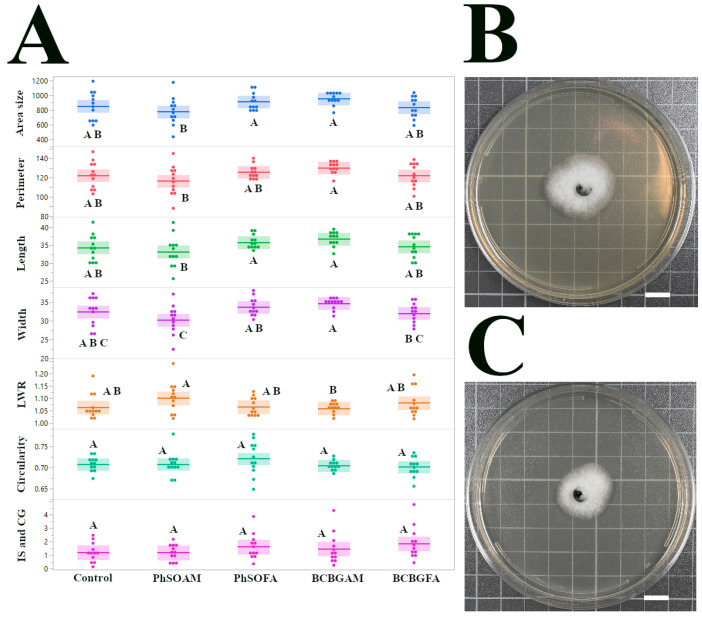
Assessment of the antifungal effects of phenolic compounds on *C. gloeosporioides* (CGH17): (**A**) Dot plots with solid lines indicating the mean and the interquartile range (25th to 75th percentiles), displaying the distribution of seven morphological traits. Statistically significant differences (*p* < 0.05) are marked by different letters above the plots, as determined by pairwise *t*-tests. Note the general lack of significant differences between treatments, indicating minimal inhibition of CGH17 by the tested compounds, except PhSOAM treatment. (**B**) Representative image of a CGH17 colony after 96 h of growth on a control (ethanol-treated) PDA plate. (**C**) Representative image of a CGH17 colony after 96 h of growth on a PDA plate treated with PhSOAM, showing minimal but statistically significant changes in colony size. White scale bars in (**B**,**C**) indicate 1 cm. *n* = 12 in all cases.

We analyzed insensitivity to all the tested compounds, and the results are shown in Figure 3, illustrating the response of *C. gloeosporioides* isolate CGH17 to the four tested compounds. Unlike the pronounced sensitivity of *Pestalotiopsis* sp. (CGH5), CGH17 exhibited minimal growth change across all treatments. Mock-treated CGH17 colonies on PDA averaged 851.18 ± 55.06 mm^2^ (SEM; *n* = 12). Treatment with PhSOAM reduced area size to 773.18 ± 54.69 mm^2^ (SEM; *n* = 12), but the difference was not significant (*p* = 0.34 versus control). Similarly, BCBGAM (950 ± 23.57 mm^2^; SEM; *n* = 12) and PhSOFA (909.65 ± 34.58 mm^2^; SEM; *n* = 12) showed no substantial effect relative to the control. While PhSOAM-treated colonies were significantly smaller than those treated with BCBGAM (*p* = 0.0056) or PhSOFA (*p* = 0.03), these differences were minor and did not reflect effective control. Colony morphology, including LWR, circularity, and the IS & CG, remained unchanged across treatments. This consistent lack of response in growth and shape shows CGH17’s strong insensitivity to all tested compounds, suggesting distinct cellular or metabolic differences from the susceptible CGH5.

### 3.3. Growth Inhibition and Altered Morphology of C. gloeosporioides Isolate CGH49

**Figure 4:**
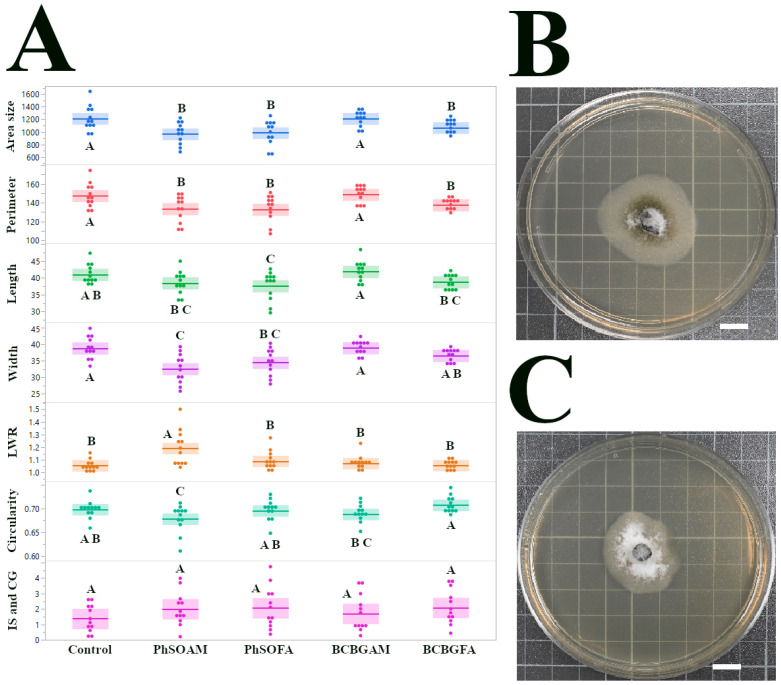
Assessment of the antifungal effects of phenolic compounds on *C. gloeosporioides* (CGH49): (**A**) Dot plots with solid lines indicating the mean and the interquartile range (25th to 75th percentiles), displaying the distribution of seven morphological traits. Statistically significant differences (*p* < 0.05) are marked by different letters above the plots, as determined by pairwise *t*-tests, with PhSOAM, PhSOFA, and BCBGFA showing partial inhibition. (**B**) Representative image of a CGH49 colony after 96 h of growth on a control PDA plate. (**C**) Representative image of a CGH49 colony after 96 h of growth on a PDA plate treated with PhSOAM, showing a reduction in colony size. White scale bars in (**B**,**C**) indicate 1 cm. *n* = 12 in all groups.

The response of *C. gloeosporioides* isolate CGH49 to test compounds is shown in Figure 4. Unlike the complete insensitivity seen in CGH17 or the potent inhibition of CGH5 by PhSOAM, CGH49 displayed a pattern of partial, yet statistically significant, growth inhibition in response to three of the four test compounds. The average colony area for the control group (CGH49 grown on control PDA) was 1212.56 mm^2^ (±56.39 mm^2^ SEM; *n* = 12). Treatment with the phenol-branched fatty amide PhSOAM resulted in a reduced mean colony area of 966.91 mm^2^ (±52.7 mm^2^ SEM; *n* = 12). Similarly, BCBGFA and PhSOFA also led to significantly smaller colony areas, averaging 985.23 mm^2^ (±56.31 mm^2^ SEM; *n* = 12) and 1065.76 mm^2^ (±26.66 mm^2^ SEM; *n* = 12), respectively. Each of these reductions was statistically significant compared to the control (pairwise *t*-tests, *p* ≤ 0.03). In contrast, BCBGAM had no discernible effect on CGH49’s growth. Reductions in colony area with PhSOAM, PhSOFA, and BCBGFA paralleled decreases in perimeter, length, and width for the PhSOAM, PhSOFA, and BCBGFA treatments. Notably, PhSOAM also induced a significant change in colony shape. The LWR for PhSOAM-treated colonies was 1.19 (±0.041 SEM), significantly higher than the control value of 1.05 (±0.013 SEM) and all other treatments (*p* < 0.0025 for all comparisons). This indicates that PhSOAM caused the colonies to grow in a more elongated fashion, similar to CGH5. These findings reveal that *C. gloeosporioides* isolate CGH49 exhibits an intermediate level of sensitivity to the tested phenolic compounds. It is neither completely insensitive, as seen with CGH17, nor as profoundly susceptible as CGH5 was to PhSOAM.

### 3.4. Comparative Antifungal Effects Across All Isolates

**Figure 5:**
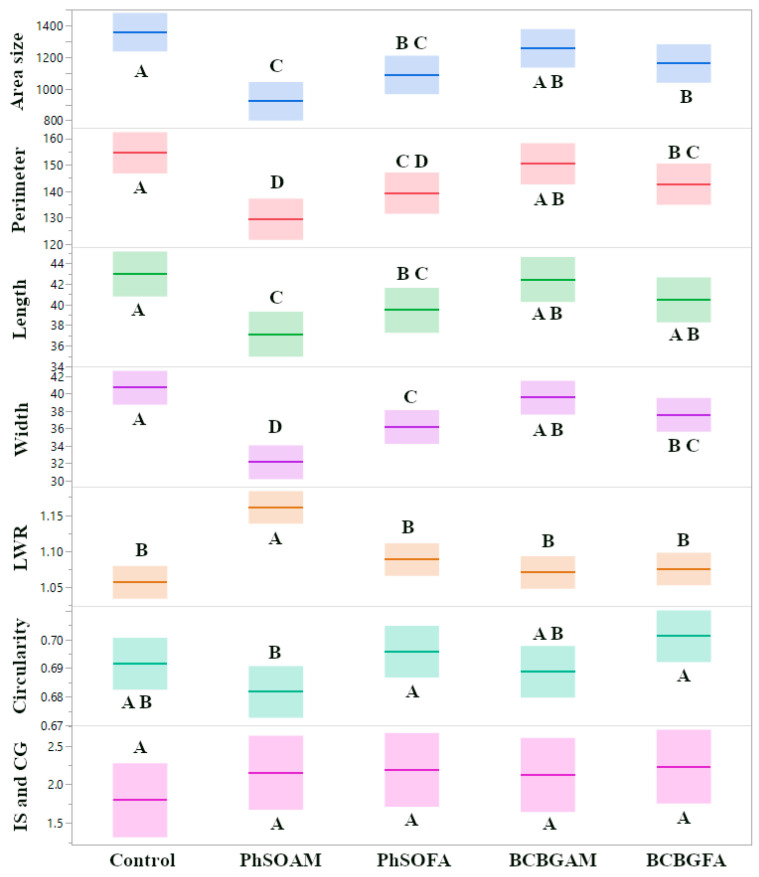
Comparative antifungal effects of phenolic branched compounds across *C. gloeosporioides* (CGH17, CGH49) and *Pestalotiopsis* sp. (CGH5). Box plots showing mean and interquartile range (25th–75th percentiles) for the seven morphological traits. Significant differences (*p* < 0.05, pairwise *t*-tests) are indicated by distinct letters above or below the plots. PhSOAM consistently reduced growth parameters and increased LWR most significantly.

Figure 5 summarizes the combined antifungal effects of the four test compounds across all three fungal isolates (CGH5, CGH17, and CGH49). Analysis of the pooled data reveals a clear hierarchy of effectiveness, with PhSOAM, a phenol-branched fatty amide, demonstrating the most potent growth inhibition. PhSOFA and BCBGFA, both phenolic branched fatty acids, exhibited intermediate efficacy, while BCBGAM, a phenolic branched fatty amide, showed minimal to no inhibitory effect. Across all isolates, the mean colony area for the control group was 1360.76 mm^2^ (±89.13 mm^2^ SEM). PhSOAM treatment significantly reduced the mean colony area to 924.29 mm^2^ (±46.53 mm^2^ SEM; *p* < 0.0001 compared to control). PhSOFA and BCBGFA also significantly reduced colony area compared to the control, resulting in mean areas of 1090.23 mm^2^ (±49.43 mm^2^ SEM; *p* = 0.0022) and 1162.1 mm^2^ (*p* = 0.0024), respectively. In contrast, BCBGAM treatment resulted in a mean colony area of 1258.01 mm^2^, which was not statistically different from the control (*p* = 0.24). Consistent with the area measurements, other size-related traits (perimeter, length, and width) followed the same pattern, with PhSOAM showing the greatest reduction, followed by PhSOFA and BCBGFA, but BCBGAM showed no significant difference from the control. Furthermore, as observed in the individual isolate analyses, PhSOAM uniquely and consistently altered colony morphology across the susceptible isolates. This was evidenced by a significantly increased LWR compared to the control and all other treatment groups (*p* < 0.0001 for all comparisons), suggesting a distinct mechanism of action (Figure 5).

### 3.5. Multivariate Analyses Uncover Complex Interplay Between Fungal Isolate Identity, Phenolic Compound Treatment, and Colony Morphology

**Figure 6:**
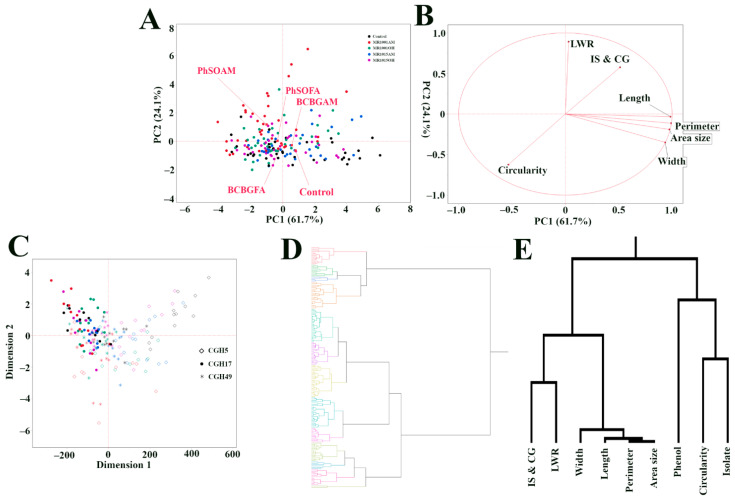
Multivariate analyses of fungal isolates, phenolic compound treatments, and colony morphology: (**A**) PCA scatter plot of all fungal colonies based on seven morphological traits, with points color-coded by treatment: control (black), PhSOAM (brown), PhSOFA (green), BCBGAM (blue), BCBGFA (purple). PC1 (61.7% variance) and PC2 (24.1% variance) are shown. (**B**) PCA biplot displaying loadings of the seven traits on PC1 and PC2; vector length reflects trait contribution, and angles indicate correlations. (**C**) MDS plot visualizing sample dissimilarities, shape-coded by isolate, along with color-code identical to (**A**): CGH5 (diamonds), CGH17 (circles), CGH49 (asterisks). (**D**) Hierarchical clustering (Ward’s method) of all colonies. The colors in the dendrogram branches represent distinct clusters identified by the analysis. (**E**) Hierarchical clustering of the seven traits, plus “Phenol” (treatment type) and “Isolate”.

To delve deeper into the relationships between fungal isolates, phenolic compound treatments, and the observed morphological variations, we employed PCA, MDS, and hierarchical clustering to provide a holistic view of the complex interactions at play. Figure 6A presents the PCA results, encompassing all seven morphological traits across all treatments and isolates. The first two PCs capture a substantial portion of the overall variance: PC1 explains 61.7%, and PC2 explains 24.1%, for a combined total of 85.8%. While the PCA plot reveals considerable overlap between the treatment groups, suggesting some shared morphological features, a trend towards separation is noticeable, particularly for the control and PhSOAM-treated samples. The PCA biplot (Figure 6B) provides insight into the relationships between the measured morphological traits. As expected, size-related traits formed a unique cluster, suggesting their inherent strong and direct correlations. LWR and IS & CG form a separate group, while circularity formed its own distinct position. This pattern suggests that these three sets of traits capture different, relatively independent traits. MDS analysis (Figure 6C) clarifies the relationships across the different experimental groups; the MDS plot revealed separation not only between the different treatment groups but also between the three fungal isolates, demonstrating the significant contribution of both inherent isolate-specific characteristics and the applied treatments to the observed morphological diversity. The hierarchical clustering of all samples (Figure 6D) identified 13 distinct clusters, showing the substantial morphological variation present within the dataset in this study. Lastly, hierarchical clustering of the traits, treatment type, and fungal isolate, using Ward’s linkage method (Figure 6E), provides a view of interrelationships among the traits. Size-related traits (area, perimeter, length, and width) form a cohesive cluster, while LWR and IS & CG form another. Intriguingly, circularity clusters closely with both “type of phenol compound treated” and “fungal isolate,” suggesting that this trait is influenced by an interaction between the inherent properties of the fungus and the specific chemical treatment it receives.

### 3.6. Machine Learning Model Selection and Feature Importance Analysis for Predicting Fungal Colony Area

To identify the best-performing model for predicting fungal colony area and to quantify the relative importance of different predictor variables, a machine learning analysis was conducted using JMP Pro 17’s “model screening” function. Models were trained to predict colony area size using the following predictors: fungal isolate (CGH5, CGH17, and CGH49), treatment type (control, PhSOAM, PhSOFA, BCBGAM, and BCBGFA), LWR, circularity, and the IS & CG. Perimeter, length, and width were excluded as predictors due to their direct relationship with area to avoid multicollinearity. Across the validation sets, a Neural Boosted model [NTanH(3)NBoost(20)] exhibited the highest average R-squared value (0.74), indicating superior predictive performance compared to other tested models, including Support Vector Machine with Radial Basis Function kernel (SVM-RBF) (0.66) [35], K-Nearest Neighbor (KNN, 0.65) [36], Bootstrap Forest (0.65) [37], Boosted Tree (0.64) [38], Decision Tree (0.63) [39], and Generalized Regression with a ridge penalty (0.62) [40]. Consequently, the Neural Boosted model was selected for further analysis. Table 1 presents the feature importance metrics for the selected Neural Boosted model. Fungal isolate emerged as the most influential predictor (Portion = 0.57), followed by treatment type (Portion = 0.32). The remaining morphological traits (LWR, IS & CG, and circularity) had comparatively minor contributions to the model’s predictive accuracy (Portions ranging from 0.029 to 0.047).

**Figure 7:**
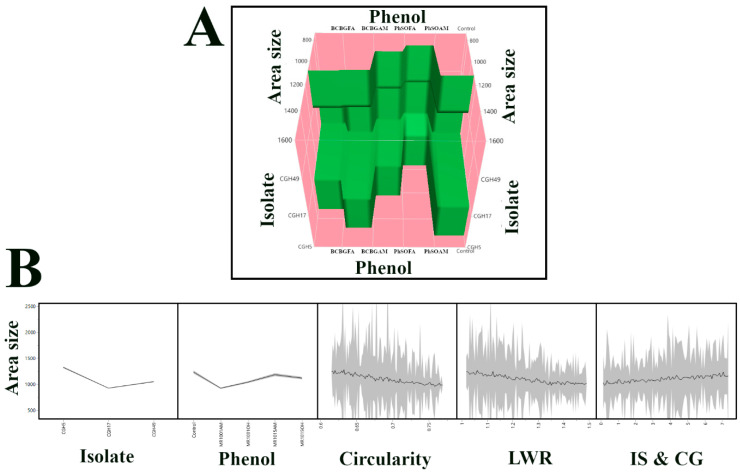
Neural Boosted model predictions of fungal colony area: (**A**) Surface plot showing the combined effects of fungal isolate and treatment on predicted colony area. (**B**) Prediction profiler plotting colony area against predictors: isolate, treatment (“Phenol”), circularity, LWR, and IS & CG. Black lines indicate predicted values; grey shading shows 95% confidence intervals, narrower for isolate and treatment than for morphological traits, reflecting greater model precision.

Figure 7A displays a surface plot generated from the Neural Boosted model, visualizing the relationship between fungal isolate, treatment type, and predicted colony area. The plot confirms that CGH5 exhibits the largest colony area under control conditions and demonstrates the consistent and pronounced reduction in area size across all isolates with PhSOAM treatment. Figure 7B shows a prediction profiler, illustrating the model’s predicted response for each predictor variable, along with confidence intervals. The narrow confidence intervals for isolate and treatment type indicate high model confidence in these predictions, while the wider confidence intervals for LWR, IS & CG, and circularity reflect their lower importance in predicting colony area due to non-linear relationships between traits.

## 4. Discussion

This study investigated the antifungal activity of four novel phenolic-branched compounds against three fungal isolates from cacao, revealing a complex spectrum of isolate-specific responses. The profound sensitivity of *Pestalotiopsis* sp. (CGH5) to PhSOAM, contrasted with the complete insensitivity of *C. gloeosporioides* isolate CGH17 and the intermediate susceptibility of isolate CGH49, underscores the critical role that fungal genetics play in determining the efficacy of antifungal agents [29]. This differential susceptibility highlights the challenge of developing broad-spectrum antifungal agents and emphasizes the need for a deeper understanding of the specific interactions between chemical compounds and fungal targets [15].

### 4.1. Structure–Activity Relationship and Sustainable Design

A clear SAR emerged from our findings. The superior performance of the phenol-branched fatty amide (PhSOAM) over its corresponding fatty acid (PhSOFA) strongly suggests that the ethylenediamine-derived amide group is crucial for potent antifungal activity. This aligns with literature demonstrating that fatty acid amides often possess enhanced membrane permeability and antimicrobial properties compared to their parent fatty acids [14,20]. While this study utilized technical-grade mixtures (preventing a strict chain-length comparison), the robust activity of soybean-derived compounds (predominantly C18) suggests that the long alkyl chains provide the necessary lipophilicity to facilitate membrane insertion, acting as an essential hydrophobic anchor for the active phenolic moiety. Critically, this study utilized compounds derived from technical-grade renewable feedstocks (e.g., brown grease). While the use of such mixtures introduces chemical complexity compared to pure synthetic reagents, it represents a strategic advantage for agricultural applications. The observed efficacy of BCBGFA/AM, despite being derived from waste streams, suggests that costly purification steps may not be necessary for field applications. This supports the viability of a “circular bioeconomy” approach in agrochemical development, where waste valorization balances efficacy with economic sustainability [41,42]. Finally, while the bio-based origin of these compounds, derived from renewable soybean oil and natural phenolic acids, suggests a potentially favorable safety profile compared to synthetic petrochemical fungicides, comprehensive safety assessments are essential. Future studies will focus on the “lead optimization” phase, specifically evaluating cytotoxicity against mammalian cells and phytotoxicity on *Theobroma cacao* tissues to ensure that these amide-based candidates are not only potent but also safe for agricultural application.

### 4.2. Morphological Disruption as a Key to Virulence Suppression

The most striking finding was the unique morphological change induced by PhSOAM. In susceptible isolates, treatment led to a significant increase in the LWR, indicating a shift towards a hyper-elongated, deregulated growth pattern. In plant pathogenic fungi, the maintenance of cell polarity and specific morphological structures (e.g., appressoria) is strictly required for host penetration and colonization [43]. The inability to maintain a coherent colony shape under PhSOAM stress suggests a disruption of the cytoskeleton or cell wall integrity, potentially involving the Spitzenkörper complex, which directs hyphal extension [44]. This hyper-elongation phenotype may also reflect an aberrant activation of the cell wall integrity signaling pathway, where fungi induce compensatory chitin synthesis and polarized growth in response to cell wall stress [45,46]. This “morphological deregulation” implies that even if the fungus is not immediately killed, its pathogenicity could be severely compromised. Previous studies have shown that agents disrupting cell wall assembly often lead to a trade-off between survival and virulence [47,48]. Thus, PhSOAM may function not just as a fungistatic agent, but as an anti-virulence compound. While ultrastructural analyses (e.g., SEM or TEM) are required to visualize the specific cellular defects, the macroscopic “hyper-elongation” phenotype observed in this study serves as a reliable high-throughput indicator of polarized growth disruption, providing a strong rationale for prioritizing this compound for detailed mechanistic studies in the future.

### 4.3. Resistance Mechanisms in C. gloeosporioides CGH17

The complete insensitivity of *C. gloeosporioides* isolate CGH17 to all tested compounds points to a robust intrinsic tolerance mechanism rather than acquired resistance, as these isolates had no prior exposure to these specific agents. This tolerance could arise from several non-mutually exclusive factors, such as the overexpression of broad-spectrum efflux pumps (ABC transporters) or enhanced metabolic detoxification capabilities [49]. It is also plausible that CGH17 possesses a more robust cell wall architecture or increased melanization. Melanin contributes to stress tolerance in *C. gloeosporioides* [50]. In melanized fungi, melanin has also been shown to bind certain antifungals and reduce their effective concentrations (e.g., *Cryptococcus*) [51]. Understanding this “intrinsic insensitivity” is crucial because *C. gloeosporioides* species complexes are notoriously difficult to control due to their high genetic diversity and metabolic plasticity [52]. Future comparative genomic analyses between the sensitive CGH49 and insensitive CGH17 isolates will be instrumental in identifying the specific molecular determinants, such as specific transporter genes or cytochrome P450 variants, that confer this tolerance.

### 4.4. A Morpho-Predictive Approach to Antifungal Screening

The machine learning analysis reinforces the biological observations, identifying fungal isolate and treatment type as the dominant predictors of fungal growth (R^2^ = 0.74). Conventional antifungal assays often rely on a single endpoint, such as the diameter of growth inhibition. Our multiparametric morphological analysis, however, provides a high-content phenotype profile that captures subtle physiological changes [53]. While this study focused on establishing the baseline biological responses to these novel bio-based compounds, future work will benchmark their efficacy against commercial synthetic fungicides (e.g., azoxystrobin or copper-based agents) to assess their relative potency. Nevertheless, the current results establish PhSOAM as a promising lead compound and demonstrate that quantitative morphological profiling combined with machine learning can effectively screen for compounds that target fungal growth polarity, offering a complementary approach to traditional lethality-based screenings.

## 5. Conclusions

This study demonstrates the potent, isolate-specific antifungal activity of a novel phenol-branched fatty amide, PhSOAM, against cacao pathogens. The compound’s efficacy is driven by a combination of its phenolic headgroup and its amide tail, and its primary mechanism appears to involve the disruption of fungal morphology and polarized growth. The complete resistance of one *C. gloeosporioides* isolate highlights the critical challenge of intraspecific variation in drug susceptibility. By integrating quantitative morphological analysis with predictive machine learning, this work establishes a powerful framework for characterizing antifungal compounds, providing deeper biological insights than standard screening methods. This approach not only identifies promising lead compounds like PhSOAM but also accelerates our understanding of their mechanism of action, paving the way for the rational design of new, effective antifungal strategies to safeguard vulnerable and economically important crops like cacao.

## Figures and Tables

**Table 1:** Feature importance in a Neural Boosted model predicting fungal colony area. This table reports feature importance from a Neural Boosted model [NTanH(3)NBoost(20)] trained to predict fungal colony area. Predictors included fungal isolate, treatment (control, PhSOAM, PhSOFA, BCBGAM, and BCBGFA), LWR, circularity, and IS & CG. Perimeter, length, and width were excluded to avoid multicollinearity with area. Importance is quantified by “Number of Splits” (frequency of use in splits), “Sum of Squares” (SS, reduction in squared error per feature), “Importance” (overall predictive contribution), and “Portion” (SS fraction of total SS, rounded to two significant digits). Higher values denote greater influence on predictions.

Trait	Number of Splits	Sum of Squares	Importance Score	Portion
Isolate	12	6,378,636.02	18	0.57
Treatment type	32	3,552,597.75	10	0.32
LWR	17	527,857.844	1	0.047
IS & CG	25	442,316.736	1	0.039
Circularity	20	323,315.082	0	0.029

## Data Availability

The original contributions presented in this study are included in the article/Supplementary Material. Further inquiries can be directed to the corresponding author.

## Supplementary Materials

The following supporting information can be downloaded at: https://www.mdpi.com/article/10.3390/jof12010033/s1, Supplementary Data S1.
